# Frontotemporal Lobar degeneration with TDP-43 presenting as progressive supranuclear palsy syndrome

**DOI:** 10.1186/s40478-025-02058-0

**Published:** 2025-07-09

**Authors:** Aya Murakami, Shunsuke Koga, Hiroaki Sekiya, Masataka Nakamura, Yusuke Yakushiji, Dennis W. Dickson

**Affiliations:** 1 Department of Neuroscience, Mayo Clinic, Jacksonville, FL USA; 2 Department of Neurology, Kansai Medical University, Osaka, Japan; 3 Department of Pathology and Laboratory Medicine, Hospital of the University of Pennsylvania, Philadelphia, PA USA

**Keywords:** Frontotemporal Lobar degeneration, TDP-43, Progressive supranuclear palsy

## Abstract

**Objective:**

Frontotemporal lobar degeneration with TDP-43 pathology (FTLD) usually presents with frontotemporal dementia, semantic aphasia or progressive nonfluent aphasia. Corticobasal syndrome and atypical parkinsonism have been occasionally reported, but progressive supranuclear palsy (PSP) syndrome (also known as Richardson syndrome (RS)) has not been reported in patients with FTLD-TDP. In this study we report clinical and pathologic characteristics of FTLD-TDP, clinically diagnosed as PSP syndrome (FTLD-TDP-PSP).

**Methods:**

We reviewed clinical information of 270 patients with FTLD-TDP from the Mayo Clinic brain bank and identified 5 patients with FTLD-TDP-PSP. As a control group, we selected ten consecutive patients of pathologically confirmed PSP with clinical presentations of PSP syndrome (PSP-RS). We compared the clinical and pathological features of FTLD-TDP-PSP and PSP-RS.

**Results:**

The most common clinical symptoms in FTLD-TDP-PSP were memory loss (100%) followed by parkinsonism (80%), early falls (60%), and behavioral variant FTD (60%). All patients with PSP-RS met the Movement Disorder Society’s criteria for probable PSP, while only one FTLD-TDP-PSP met the probable PSP. Two of the five patients with FTLD-TDP-PSP had moderate or severe neuronal loss in the substantia nigra and one had moderate or severe neuronal loss in the putamen and globus pallidus.

**Conclusion:**

A small subset of patients with FTLD-TDP can, in rare instances, present with symptoms associated with PSP. Therefore, FTLD-TDP may be considered in differential diagnosis, especially in patients who do not meet the diagnostic criteria. Our findings emphasize the need for further clinical and biomarker studies of FTLD-TDP.

**Supplementary Information:**

The online version contains supplementary material available at 10.1186/s40478-025-02058-0.

## Introduction

Frontotemporal lobar degeneration with TDP-43 pathology (FTLD-TDP) is pathologically characterized by neuronal loss and gliosis in the frontal and temporal lobes as well as TDP-43 positive neuronal cytoplasmic inclusions (NCI), and variable dystrophic neurites (DN) and neuronal intranuclear inclusions [24]. It typically presents with frontotemporal dementia, semantic aphasia, logopenic progressive aphasia or frontotemporal dementia with or without motor neuron disease [15, 34]. Atypical parkinsonism [22] and corticobasal syndrome (CBS) have also been reported in patients with FTLD-TDP [18, 26]; however, patients presenting with the progressive supranuclear palsy (PSP) syndrome are uncommon in FTLD-TDP.

PSP often presents with postural instability, falls, vertical gaze palsy, and levodopa-unresponsive parkinsonism, and this syndrome has been referred to as Richardson syndrome (RS), recognizing that there are other clinical presentations of PSP, such as CBS and parkinsonism. The most common pathology in patients with PSP syndrome is PSP, but occasionally corticobasal degeneration can mimic PSP syndrome. In a previous study, we characterized clinical presentations of upper-motor-neuron-predominant motor neuron disease with or without FTLD-TDP, and we found that a subset of patients presented with PSP syndrome [30]. In such patients, it is thought that clinical features of motor neuron disease can mimic PSP syndrome. It remains unclear whether FTLD-TDP without motor neuron disease can also present with PSP syndrome. The present study aimed to determine the frequency of patients who were clinically diagnosed with PSP, although their underlying pathology was confirmed FTLD-TDP (FTLD-TDP-PSP) and to characterize their clinical and pathological features, focusing on neuronal loss and TDP-43 pathology. To characterize the clinical features of FTLD-TDP-PSP, we compared them to patients with PSP syndrome and pathologically confirmed PSP (PSP-RS).

## Methods

### Case selection

The present study included 270 patients with a pathological diagnosis of FTLD-TDP from the Mayo Clinic brain bank for neurodegenerative disorders accessioned between 2000 and 2022. Cases were identified by searching the brain bank database for entries with a pathological diagnosis of FTLD-TDP.

**Fig. 1:**
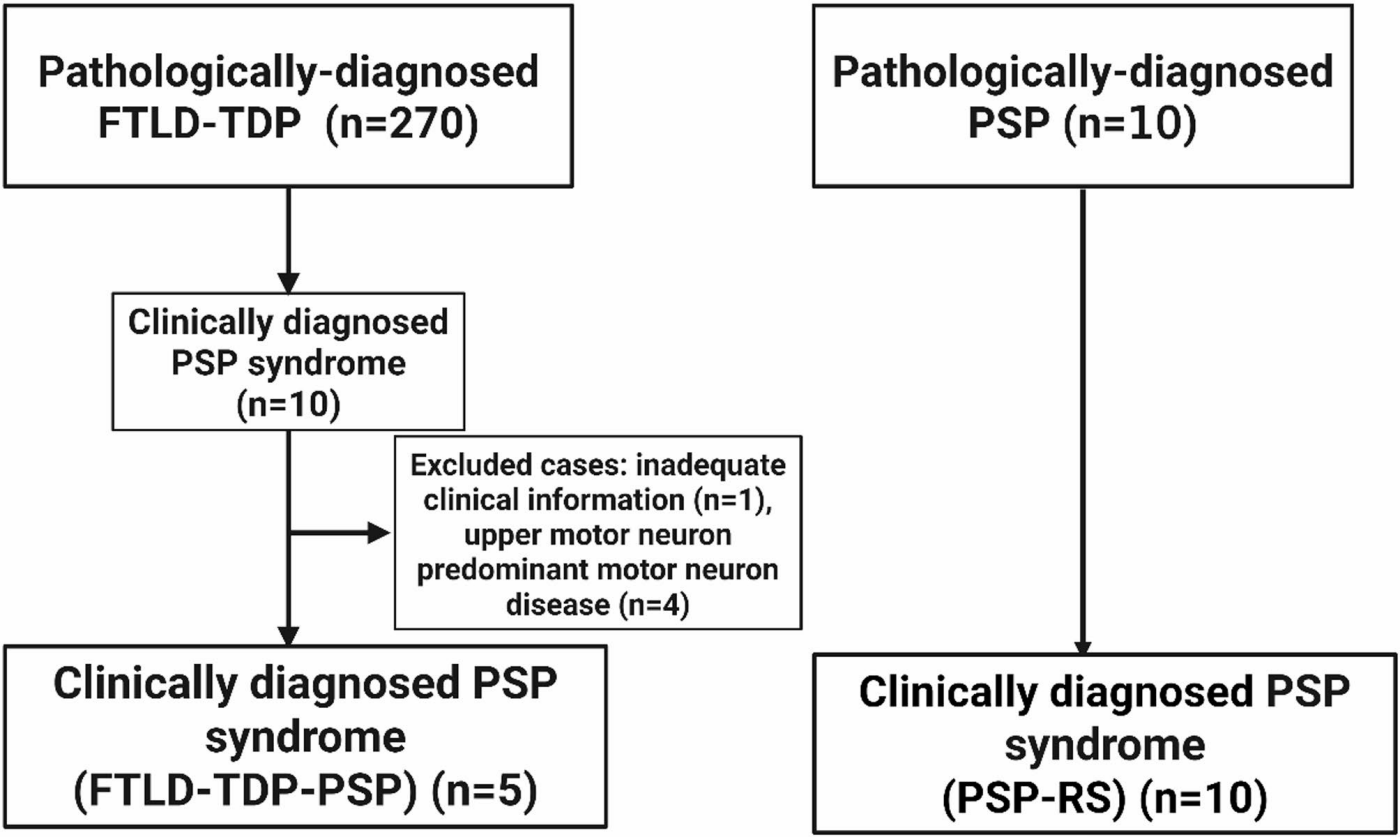
Flow chart of study design. FTLD-TDP = frontotemporal lobar degeneration with TDP-43; FTLD-TDP-PSP = frontotemporal lobar degeneration with TDP-43, clinically diagnosed as progressive supranuclear palsy syndrome; PSP-RS = progressive supranuclear palsy with PSP syndrome; PSP = progressive supranuclear palsy; RS = Richardson syndrome

Upon reviewing medical records, ten of these patients had a final clinical diagnosis of PSP. Cases were excluded if they had a pathological diagnosis of upper-motor-neuron-predominant motor neuron disease (*n* = 4) or if they lacked adequate medical records (*n* = 1). The final cohort consisted of 5 patients with FTLD-TDP-PSP. As a control group, we selected 10 consecutive patients who had a clinical diagnosis of PSP and were pathologically confirmed as PSP; these cases were defined as PSP-RS. The study design is shown in Fig. 1.

### Clinical assessment

Two neurologists (A.M., S.K.) abstracted the following information from medical records collected throughout the course of the disease and entered the information into a database. The following variables were assessed: sex, age at symptomatic onset, age at death, family history of neurologic disease, clinical diagnoses, initial symptoms (i.e., motor dysfunction or cognitive dysfunction), signs and symptoms during the disease course and their timing, as well as neurologic findings as documented by a neurologist or movement disorder specialist. For each patient, a particular clinical symptom or sign was considered present if specifically noted in the medical records. If clinical symptoms or signs were not described, they were considered to be absent for the purpose of analysis. The following symptoms and neurologic signs were abstracted from medical records: parkinsonism, bradykinesia, axial/limb rigidity, orobuccal or limb apraxia, myoclonus, dystonia, aphasia, falls, early falls (defined as occurring within 3 years of symptomatic onset), ocular motor dysfunction, and memory loss, pseudobulbar affect, and features of behavioral variant frontotemporal dementia (bvFTD), including behavioral disinhibition, apathy or inertia, loss of sympathy or empathy, perseverative or compulsive/ritualistic behavior, and hyperorality or dietary changes. Response to levodopa therapy was recorded as good or no good. The Movement Disorder Society’s criteria for PSP [12] were used for the diagnosis of FTLD-TDP-PSP and PSP-RS. When available the neuropsychological profile was reviewed and data from brain MRI or CT were recorded. The clinical information was obtained from medical records, pathology records summarizing the clinical history and the CurePSP brain bank questionnaire filled out by the family or someone with close knowledge of the patient [25]. Given the retrospective nature of the study, the quality of available medical records was variable from case to case.

### Neuropathologic assessment

Most of the brains were received with the left hemibrain fixed in 10% formalin; the right hemibrain frozen at -80 °C. All cases underwent systematic and standardized sampling with neuropathologic evaluation by an experienced neuropathologist (DWD). Paraffin-embedded 5-µm thick sections mounted on glass slides were stained with hematoxylin and eosin (H&E) and thioflavin S. H&E-stained sections from the substantia nigra, corticobulbar tract in the cerebral peduncle, hippocampus with parahippocampal gyrus, subthalamic nucleus, putamen, globus pallidus, amygdala, nucleus basalis of Meynert, superior frontal gyrus, middle frontal gyrus, inferior parietal gyrus, superior temporal gyrus, occipital temporal gyrus, and motor cortex were reviewed by three observers (A.M., S.K., D.W.D.) to assess neuronal loss on four-point semiquantitative scores (0 = absent; 1 = mild; 2 = moderate; 3 = severe) as previously reported [17, 30].

To confirm the neuropathological diagnosis of FTLD-TDP, immunohistochemistry for phospho-TDP43 (pTDP-43) (pS409/410, mouse monoclonal, 1:5000, Cosmo Bio, Tokyo, Japan) or TDP-43 (MC2085, 1:3000, gift from Dr. Leonard Petrucelli, Mayo Clinic, Jacksonville, Florida) was performed on sections of mid-frontal cortex, superior and middle temporal cortex, parietal cortex, anterior cingulate gyrus, amygdala, striatum and globus pallidus, hippocampus with parahippocampal gyrus, and midbrain including substantia nigra. To exclude coexisting tauopathies, such as PSP, corticobasal degeneration [7, 11, 23], immunohistochemistry for phospho-tau (CP13, Ser202, mouse monoclonal, 1:1000, from the late Dr Peter Davies, Feinstein Institute, North Shore Hospital, NY) was performed on sections of mid-frontal cortex, superior and middle temporal cortex, parietal cortex, anterior cingulate gyrus, amygdala, striatum and globus pallidus, hippocampus with parahippocampal gyrus, subthalamic nucleus, midbrain, pons, and cerebellum.

Braak neurofibrillary stage and Thal amyloid phase were assigned based upon lesion density and distribution with thioflavin S fluorescent microscopy according to published criteria [3, 21, 37]. Alzheimer’s type neuropathologic change was based on the consensus criteria for the neuropathologic diagnosis of Alzheimer’s disease [13]. Lewy-related pathology was assessed by α-synuclein immunohistochemistry (NACP, rabbit polyclonal, 1:3000; Mayo Clinic, Jacksonville, Florida) [28] in the amygdala, basal forebrain, brainstem, cingulate gyrus, and temporal lobe, and classified as the brainstem, limbic and diffuse types. Neuronal inclusions were detected using p62/sequestosome-1 immunohistochemistry (p62 lck ligand, mouse monoclonal, 1:250, BD Transduction, Franklin Lakes, New Jersey) in cerebellum [1], which have been described in subjects with *C9ORF72* hexanucleotide repeats.

### Assessment of TDP-43 pathology

All slides were reviewed simultaneously by two observers (A.M., S.K.) who agreed on the presence of TDP-43 immunoreactivity, defined as NCI, DN, glial cytoplasmic inclusion, or neuronal intranuclear inclusions, in the midbrain, basal ganglia, amygdala, hippocampus, superior temporal cortex, middle frontal cortex, and motor cortex. The severity of TDP-43 pathology was graded semi-quantitatively on a five-point scale (0 = absent, 1 = rarely, 2 = mild, 3 = moderate, 4 = severe) as previously reported [29].

### Assigning FTLD-TDP subtype

Each case was classified into an FTLD-TDP subtype based on the criteria [24]. Type A is characterized by abundant NCI and short DN in neocortical layer II and the presence of lentiform neuronal intranuclear inclusions. Type B is characterized by abundant NCI and few DN in all cortical layers. Type C is characterized by abundant long DN and few NCI in all cortical layers. Cases were assigned Type A, Type B or Type C.

### Statistical methods

All statistical analyses were performed using R version 4.0.5 (The R Foundation for Statistical Computing, Vienna, Austria) and EZR (Saitama Medical Center, Jichi Medical University Saitama, Japan), which is a graphical interface for R [16]. A Fisher’s exact test was performed for group comparisons of categorical data, as appropriate. Analysis of t-test or Mann–Whitney U test was used for analyses of continuous variables, as appropriate. p-values < 0.05 were considered statistically significant. Hierarchical cluster analysis using Euclidean distance and average linkage clustering was performed on patients and region-specific variables reflecting the severity of the neuronal loss or TDP-43 pathology as previously reported [30].

## Results

### Demographic and pathological features

Table 1 summarizes the demographic and pathologic features of FTLD-TDP-PSP and PSP-RS patients. The average age at onset in FTLD-TDP-PSP patients was 66 ± 4 years ranging from 61 to 70, and the average disease duration was 8.2 ± 6.5 years ranging from 1.3 years to 17.7 years. Family history of amyotrophic lateral sclerosis was noted in 40% of FTLD-TDP-PSP patients, and one patient had a family history of dementia. There were no significant differences in the proportion of male/female, age at onset, disease duration to death between FTLD-TDP-PSP and PSP-RS. Pathologic features, including brain weight, Braak neurofibrillary tangle stage, Thal amyloid phase, and Lewy-related pathology did not differ between the two groups. One patient had *C9ORF72* repeat expansion based upon p62 immunohistochemistry in the cerebellum.

**Table 1:** Demographic and pathological features of FTLD-TDP-PSP and PSP-RS

Clinical features	FTLD-TDP-PSP	PSP-RS	*P*-value
Number	5	10	
Male	40% (2)	40% (4)	
Age at onset, years	66 ± 4	67 ± 8	0.77
Age at death, years	74 ± 5	73 ± 8	0.89
Disease duration, years	8.2 ± 6.5	6.7 ± 2	0.49
**Pathological features**			
Brain weight, grams	934 ± 222	1101 ± 202	0.17
Braak stage	II (II, II)	III (II, III)	0.70
Thal phase	1 (0, 3)	1 (0, 1)	0.60
Hippocampal sclerosis	40% (2)	0% (0)	0.08
Lewy-related pathology	0% (0)	10% (1)	1.00

FTLD-TDP-PSP = frontotemporal lobar degeneration with TDP-43, clinically diagnosed as progressive supranuclear palsy syndrome; PSP-RS = progressive supranuclear palsy presenting with progressive supranuclear palsy syndrome; Data are displayed as n (%), mean ± SD, and median (25th, 75th range)

### Clinical features of FTLD-TDP-PSP and comparing FTLD-TDP-PSP versus PSP-RS

Supplemental Table lists the frequency of clinical features in FTLD-TDP-PSP and PSP-RS. The most common clinical symptoms were parkinsonism (80%) and memory loss (80%), followed by early falls (60%), and bvFTD symptoms (60%). No patients with FTLD-TDP-PSP met criteria for bvFTD [32]. Among those with parkinsonism, 40% showed asymmetrical symptoms. Two patients were prescribed antidepressants after the onset of parkinsonism and clinical diagnosis of PSP, suggesting that drug-induced parkinsonism was unlikely.

We next compared the clinical features of FTLD-TDP-PSP with PSP-RS (Table 2). Eight patients with PSP-RS met the Movement Disorder Society criteria for probable PSP with Richardson’s syndrome and two patients met criteria for PS with predominant parkinsonism, while only one FTLD-TDP-PSP patient met criteria for probable PSP with Richardson’s syndrome. Two FTLD-TDP-PSP met criteria for suggestive PSP with predominant postural instability. Of note, all PSP-RS patients had eye movement dysfunction defined by the Movement Disorder Society’s criteria for PSP (i.e., vertical supranuclear gaze palsy and slow velocity of vertical saccades), but only 2 of 5 FTLD-TDP-PSP patients had this sign, one of whom had a *C9ORF72* repeat expansion. Other features, such as the frequency of parkinsonism, early falls, bvFTD, aphasia, L-dopa responsiveness, memory loss, and apraxia, did not significantly differ between the two groups.

**Table 2:** Clinical features in FTLD-TDP-PSP vs. PSP-RS

	FTLD-TDP-PSP	PSP-RS	*P*-value
Parkinsonism	80% (4)	100% (10)	0.33
Early falls	60% (3)	80% (8)	0.56
Oculomotor Dysfunction	40% (2)	100% (10)	0.02
bvFTD	60% (3)	30% (3)	0.33
Aphasia	40% (2)	30% (3)	1
L-dopa responsiveness	25% (1/4)	80% (8)	0.09
Memory loss	80% (4)	80% (8)	1
Apraxia	0% (0)	20% (2)	1
MDS-PSP criteria			< 0.01
Probable PSP	20% (1)	100% (10)	
Possible PSP	0% (0)	0% (0)	
Suggestive PSP	40% (2)	0% (0)	
Neuroimaging	*n* = 5	*n* = 8	
Midbrain atrophy	20% (1)	50% (4)	0.56
Frontotemporal atrophy	40% (2)	12.5% (1)	0.51
Duration final imaging study from death, months	68 ± 19	40 ± 18	0.52

bvFTD = behavioral variant frontotemporal dementia; FTLD-TDP-PSP = frontotemporal lobar degeneration with TDP-43, clinically diagnosed as progressive supranuclear palsy syndrome; PSP-RS = progressive supranuclear palsy presenting with progressive supranuclear palsy syndrome

Imaging was performed using MRI in all FTLD-TDP-PSP cases, and in the PSP-RS group, 8 cases underwent MRI and 2 underwent CT. Data are displayed as n (%), mean ± SD, and median (25th, 75th range)

### Neuropathological characteristics in FTLD-TDP-PSP

**Fig. 2:**
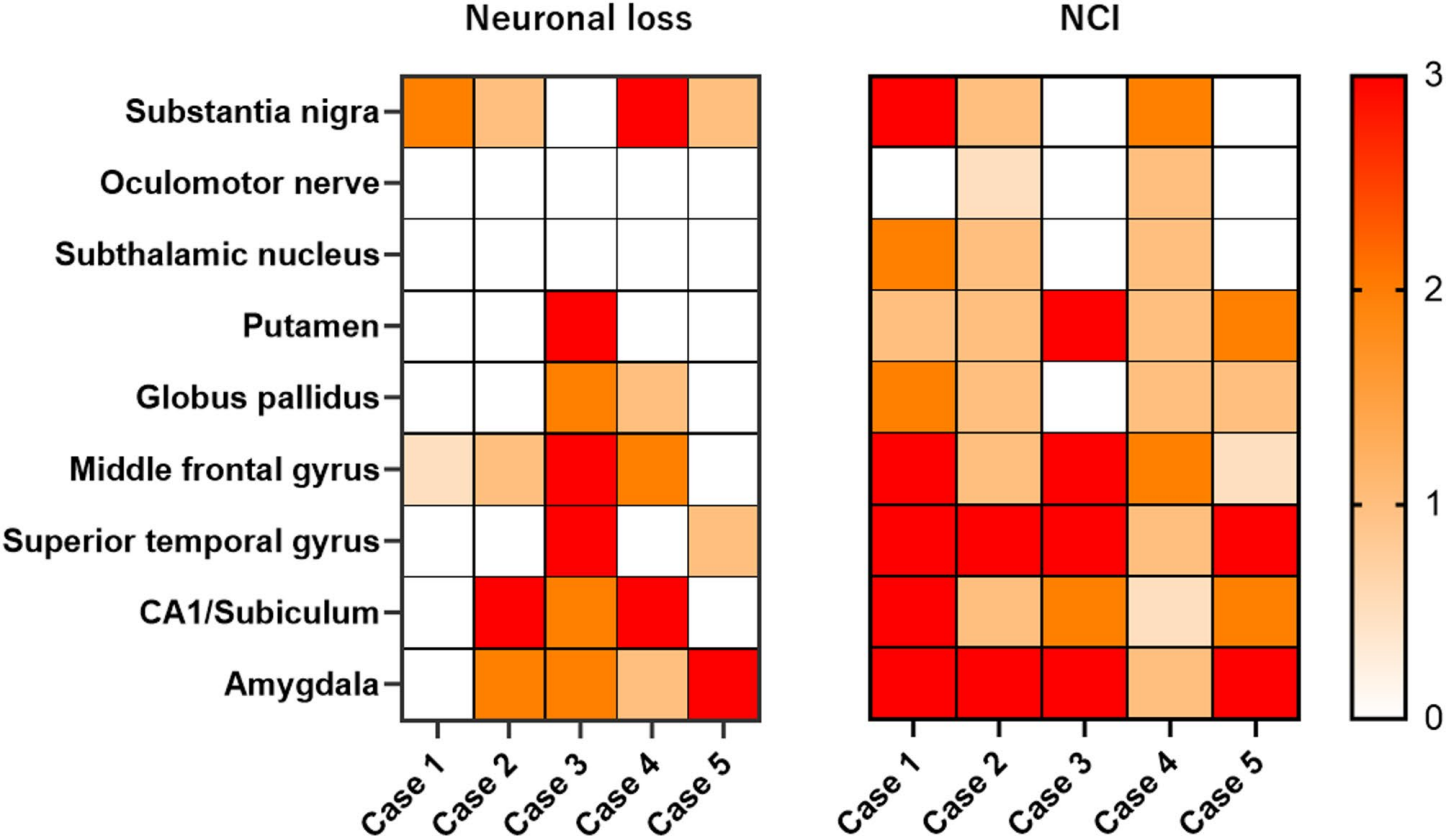
Heatmap based on neuronal loss and neuronal cytoplasmic inclusions in 5 cases of FTLD-TDP-PSP. The heatmap reflects the severity of neuronal loss and density of neuronal cytoplasmic inclusions in each brain region. The color scale is on the right. According to the Movement Disorder Society progressive supranuclear palsy (PSP) diagnostic criteria, Case 1 fulfilled the criteria for probable PSP, Cases 2 and 3 for suggestive PSP, and Cases 4 and 5 did not meet the diagnostic criteria. NCI = TDP-43-positive neuronal cytoplasmic inclusions

We examined the distribution of neuronal loss in eight brain regions of FTLD-TDP-PSP (Fig. 2). As shown in the heatmap, four patients had neuronal loss in the substantia nigra (Cases 1, 2, 4, and 5), and two patients (Cases 1 and 4) had moderate or severe neuronal loss. The remaining one patient (Case 3) did not have neuronal loss in the substantia nigra, but did have severe neuronal loss in the putamen and a moderate neuronal loss in the globus pallidus. No FTLD-TDP-PSP had significant neuronal loss in the subthalamic nucleus, where most PSP-RS patients had moderate to severe neuronal loss.

**Fig. 3:**
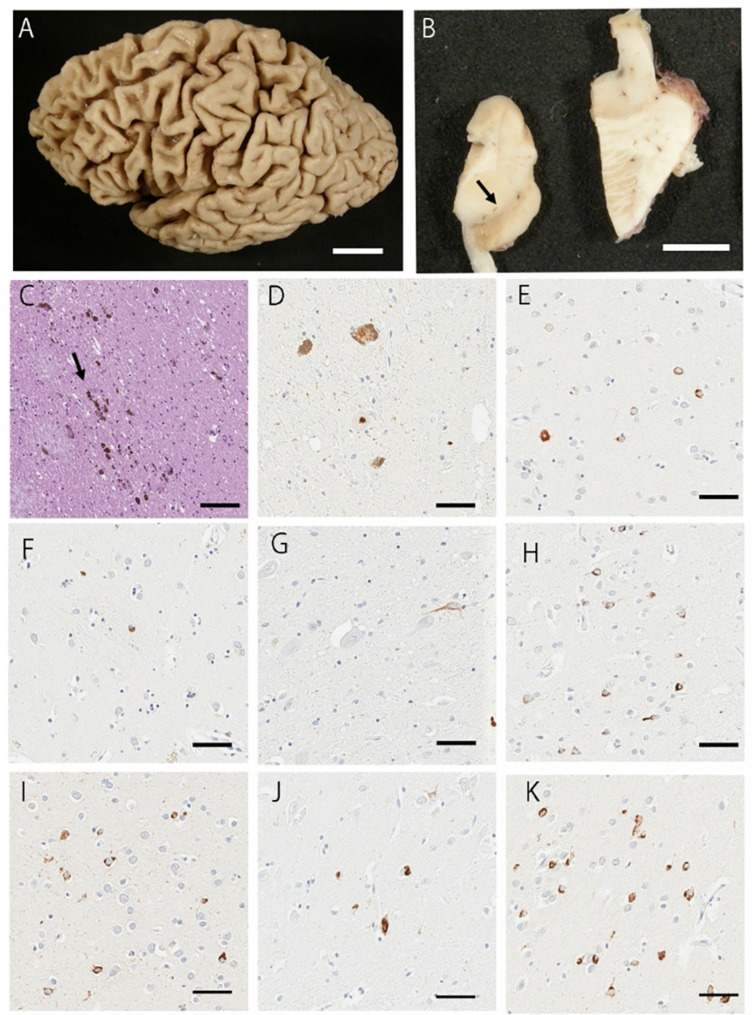
Representative macroscopic and histopathologic images. **(A)** The brain shows frontotemporal atrophy. **(B)** The substantia nigra has reduced pigment (arrow). **(C)** The H&E staining of the substantia nigra shows a moderate neuronal loss. Free melanin is observed (arrow). (**D**-**K**) pTDP43-positive NCIs and glial cytoplasmic inclusions are observed in the substantia nigra **(D)**, globus pallidus **(E)**, putamen **(F)**, subthalamic nucleus **(G)**, middle frontal gyrus **(H)**, superior temporal gyrus **(I)**, subiculum **(J)**, and amygdala **(K)**. **B**: H&E staining; **D-K**: pTDP43 immunostaining. Scale bars: A = 3 cm; B = 1 cm; C-K = 50 μm

The severity of TDP-43 pathology in eight brain regions is summarized in Fig. 2. Two patients (Cases 1 and 4) had more than moderate TDP-43 pathology in the substantia nigra. Three patients had NCI in the subthalamic nucleus (Cases 1, 2 and 4). All cases had NCI in the putamen. The FTLD-TDP subtypes of the cases included 3 cases (Cases 1, 2, and 4) with Type A, who all had NCIs, and 2 cases (Cases 3 and 5) with Type B. Representative macroscopic and histopathologic images of FTLD-TDP-PSP cases are shown in Fig. 3. Detailed neuropathological findings for each FTLD-TDP-PSP case are provided in Supplementary Data.

## Discussion

The aim of this study was to identify patients with FTLD-TDP-PSP and characterize their clinicopathological features. In our brain bank, only 5 of 270 patients (2%) with FTLD-TDP had a final clinical diagnosis of PSP, consistent with FTLD-TDP-PSP, without coexisting primary tauopathies. A review of clinical features revealed that vertical gaze palsy was uncommon in patients with FTLD-TDP-PSP, with only one of the five meeting the rigorous diagnostic criteria for probable or possible PSP. Neuropathological evaluation revealed that three patients had moderate to severe neuronal loss in the substantia nigra or putamen, which may represent the neuropathologic correlate of parkinsonism. All three also had TDP-43 pathology in the substantia nigra. These findings suggest that although rare, FTLD-TDP can present with symptoms associated with PSP and is often associated with neuronal loss and TDP-43 pathology in the striatonigral system.

The findings of this study expand the understanding of the clinical presentations of FTLD-TDP by identifying FTLD-TDP-PSP as a novel clinical phenotype. TDP-43 pathology has been implicated as an underlying pathology of PSP syndrome, particularly in studies on *C9ORF72* repeat expansions. A review of six different genetic studies that evaluated *C9ORF72* repeat expansions in patients with PSP syndrome [2, 10, 19, 20, 31, 35, 40] identified three FTLD-TDP-PSP cases with abnormal *C9ORF72* repeat expansions among 240 patients. All three showed parkinsonism and eye movement dysfunction, including vertical supranuclear palsy or gaze palsy, features suggestive of RS. Additionally, two patients had frontal lobe syndrome. Imaging studies revealed midbrain atrophy in one patient and frontotemporal lobar atrophy in another. Similarly, in the present study, the FTLD-TDP-PSP patient with a *C9ORF72* repeat expansion exhibited downward gaze palsy and square wave jerks. Although the genetic findings strongly suggest an association with TDP-43 pathology, autopsy confirmation was not available in these patients. There have also been reports of patients with non-pathogenic progranulin variants presenting with PSP [6, 27]. While TDP-43 pathology was suspected as a possible underlying pathology in these cases, autopsy was not performed, and it remains unclear whether the underlying pathology was tau or TDP-43. Given that PSP pathology coexisting with TDP-43 pathology has been reported [33], the possibility of concomitant tau pathology in these patients cannot be excluded. The present study is the first to demonstrate that patients with FTLD-TDP can present with PSP syndrome.

Comparison of clinical features between FTLD-TDP-PSP and PSP-RS provided some clues that may help distinguish between the two. One of the key differences was the frequency of vertical gaze palsy, which was lower in FTLD-TDP-PSP than PSP-RS. Only one FTLD-TDP-PSP patient met criteria for probable PSP, while all ten PSP-RS patients met the criteria for probable PSP. Regarding imaging findings, midbrain atrophy was noted in only 20% of FTLD-TDP-PSP patients compared to 50% of PSP-RS patients. In contrast, frontotemporal atrophy was more frequent in FTLD-TDP-PSP than PSP-RS. Although these findings were not statistically significant due to the small sample size, these imaging features might be helpful in differentiating FTLD-TDP-PSP from PSP-RS.

The neuropathologic substrate of PSP syndrome in FTLD-TDP remains unclear. The substantia nigra has been the most investigated brain region that is associated with parkinsonism. More than moderate neuronal loss in the substantia nigra and the putamen is associated with parkinsonism [8]. In FTLD-TDP, patients presenting with parkinsonism have also been found to have neuronal loss of the substantia nigra [36, 39]. Given that the putamen plays a key role in both the direct and indirect pathways of the basal ganglia, this lesion may contribute to parkinsonism [4]. In our study, three out of five FTLD-TDP-PSP patients had moderate or severe neuronal loss in the substantia nigra or putamen. Interestingly, a recent study demonstrated that a subset of patients with FTLD-TDP have parkinsonism but lack significant neuronal loss in the substantia nigra. Instead, the severity of TDP-43 pathology in the substantia nigra correlated with parkinsonism [9]. We found TDP-43-positive NCI in the substantia nigra in 3 of 5 cases of FTLD-TDP-PSP. Nevertheless, the pathological substrate of parkinsonism in one of the cases (Case 5) remains unknown, since there was neither neuronal loss nor TDP-43 pathology in the substantia nigra. Taken together, both neuronal loss and TDP-43 pathology in the substantia nigra may contribute to parkinsonism. Vertical gaze palsy is one of the core features of RS, and is a key differential feature for FTLD-TDP-PSP and PSP-RS; however, the pathological correlates of this sign were not identified. Although the brain regions responsible for vertical gaze palsy are not fully understood, no significant neuronal loss or TDP-43 pathology was observed in the tegmentum or subthalamic nucleus—regions typically affected in PSP-RS and considered to be involved in oculomotor dysfunction [5, 14, 38]—in our FTLD-TDP-PSP cases. This may partly explain the lower frequency of oculomotor symptoms in FTLD-TDP-PSP cases.

There are several limitations to the present study. First, we were unable to assess the laterality of pathology because only one hemi-brain was available for the pathologic assessment in all but one case. Second, the retrospective nature of this brain bank study limited the availability of clinical information in some cases. To better evaluate potential correlation between neuronal loss, TDP-43 pathology, and clinical features associated with antemortem diagnoses, it will be important to evaluate patients who undergo autopsy as part of prospective clinical studies. Third, the small sample size limited our ability to identify significant differences in clinicopathologic features between clusters. A larger cohort may help define distinct clinicopathologic subtypes of FTLD-TDP-PSP.

In conclusion, the present study demonstrates that FTLD-TDP pathology can present with symptoms resembling PSP. Therefore, FTLD-TDP should be considered in the differential diagnosis of PSP syndrome, particularly in patients who do not fulfill the criteria for clinically probable PSP syndrome. Our findings expand the clinical spectrum of FTLD-TDP and emphasize the need for further clinical, biomarker, and genetic studies of FTLD-TDP-PSP.

## Electronic supplementary material

Below is the link to the electronic supplementary material.

Supplementary Material 1

Supplementary Material 2

## Data Availability

Data that support the findings of this study are available from the corresponding author upon request.
