# Supplementary material for: Frontotemporal Lobar degeneration with TDP-43 presenting as progressive supranuclear palsy syndrome
Source: Acta Neuropathol Commun. 2025 Jul 9;13:151. doi: 10.1186/s40478-025-02058-0 (PMC12239482; doi:10.1186/s40478-025-02058-0)
Supplement: Supplementary file 1 — Supplementary Material 1 [file 40478_2025_2058_MOESM1_ESM.docx]

Supplementary Data. Neuropathological findings of FTLD-TDP-PSP cases

Case 1

The fixed left hemibrain weighed 630 grams. Macroscopic findings revealed cortical atrophy in the frontal lobe. The temporal, parietal, occipital lobes and medial temporal lobes were free of atrophy. Sequential sections revealed the hippocampal formation and amygdala were both free of atrophy. The subthalamic nucleus was unremarkable. Horizontal sections of the midbrain, pons and medulla were free of obvious pathology. The substantia nigra showed decreased pigmentation. The cerebellar sections showed no unusual features.

On hematoxylin and eosin-stained sections, mild neuronal loss and gliosis were evident in the frontal cortex. There was no hippocampal sclerosis. The basal ganglia and subthalamic nucleus had no neuronal loss. The substantia nigra had moderate neuronal loss.

Thioflavin-S fluorescent microscopy showed no senile plaques (SP) or neurofibrillary tangles (NFT) in the neocortex. The entorhinal and perirhinal cortices had a few SP and NFT in layer II and in lower cortical layers with thioflavin-S fluorescent microscopy.

Immunohistochemistry for TDP-43 revealed neuronal cytoplasmic inclusions (NCI), sparse dystrophic neurites, glial cytoplasmic inclusions (GCIs), and neuronal intranuclear inclusions (NII) in the frontal and temporal cortex. NCI and GCI were present in substantia nigra. TDP-43 pathology was present in all cortical layers, a pattern consistent with FTLD-TDP type A.

Phospho-tau immunohistochemistry revealed numerous argyrophilic grains, pretangles, tau-positive granular fuzzy astrocytes, thorn-shaped astrocytes in the inferior temporal gyri. These findings are consistent with argyrophilic grain disease stage1.

Case 2

The fixed left hemibrain weighed 440 grams. Macroscopic findings revealed diffuse mild cortical atrophy. Sequential sections revealed moderate enlargement of the anterior and temporal horns. The hippocampal formation and amygdala showed mild atrophy. Basal ganglia and the subthalamic nucleus were unremarkable. Horizontal sections of the midbrain, pons and medulla were free of obvious pathology. The substantia nigra had decreased pigmentation. On hematoxylin and eosin-stained sections, neuronal loss and gliosis were evident in the frontal cortex. Hippocampal sclerosis was observed. The subthalamic nucleus had no neuronal loss. The substantia nigra had mild neuronal loss with extraneuronal neuromelanin and gliosis, but no Lewy bodies were identified.

The thioflavin-S fluorescent microscopy demonstrated SP and a few NFT. The hippocampus had only a few NFT, but no SP in the pyramidal or the molecular layer of the dentate fascia.

Immunohistochemistry for TDP-43 revealed sparse dystrophic neurites and NCI. Only very rare NIIs were detected in the neocortex. The putamen, globus pallidus, and substantia nigra had sparse NCI and a few dystrophic neurites.

P62 immunohistochemistry revealed inclusions in the cerebellum. Phosphorylated tau immunohistochemistry did not reveal tufted astrocytes, and no typical features of PSP were observed.

Case 3

The fixed left hemibrain weighed 320 grams. Macroscopic findings revealed severe cortical atrophy, most prominent in the frontal and temporal poles, as well as in the precentral gyrus. The medial temporal lobe had mild to moderate atrophy. Sequential sections revealed marked enlargement of the anterior and temporal horns. The cortical gray mantle was markedly thinned in the frontal, temporal and parietal lobes. The subthalamic nucleus was unremarkable.

Horizontal sections of the midbrain, pons and medulla revealed marked atrophy of the cerebral peduncle. There was visible pigmentation in the substantia nigra. The cerebellar sections showed no unusual features.

On hematoxylin and eosin-stained sections, severe neuronal loss and gliosis were evident in the frontal and temporal cortex. The parietal lobes had neuronal loss and gliosis. The hippocampus had acute infarction in the CA1 sector of the hippocampus and chronic neuronal loss and gliosis in the subiculum. The subthalamic nucleus had no neuronal loss. The substantia nigra had no significant neuronal loss.

Thioflavin-S fluorescent microscopy showed sparse SP, but no NFT in the neocortex. There was no SP or NFT in pyramidal layer of Ammon's horn or the dentate fascia. There was sparse SP and sparse NFT in layer II and in lower cortical layers with thioflavin S fluorescent microscopy.

Immunohistochemistry for TDP-43 revealed many NCI in all cortical layers. The putamen and caudate nucleus had many NCIs. These findings were consistent with FTLD-TDP type B.

Phosphorylated tau immunohistochemistry did not reveal tufted astrocytes.

Case 4

The fixed left hemibrain weighed 430 grams. Macroscopic findings revealed severe cortical atrophy in the frontal and temporal lobes (Fig. [3](https://actaneurocomms.biomedcentral.com/articles/10.1186/s40478-023-01611-z#Fig1)A) The medial temporal lobe also had moderate atrophy. Sequential sections revealed marked enlargement of the anterior and temporal horns. The hippocampal formation and amygdala had mild atrophy. Basal ganglia

showed atrophy of the head of the caudate nucleus. The thalamus had a marked atrophy. The subthalamic nucleus was unremarkable. Horizontal sections of pons had marked atrophy. The substantia nigra had decreased pigmentation (Fig. [3](https://actaneurocomms.biomedcentral.com/articles/10.1186/s40478-023-01611-z#Fig1)B). The cerebellar sections showed no unusual features.

On hematoxylin and eosin-stained sections, moderate neuronal loss and gliosis were evident in the frontal cortex. There was severe neuronal loss and gliosis in CA1 and the subiculum, consistent with hippocampal sclerosis. The subthalamic nucleus had no neuronal loss. The substantia nigra had moderate patchy neuronal loss with extraneuronal neuromelanin and gliosis, but no Lewy bodies were identified.

Thioflavin-S fluorescent microscopy showed no SP or NFT in the neocortex. The entorhinal and perirhinal cortices showed superficial spongiosis and gliosis with no SP, and only a few NFT in layer II, but not in deeper layers with thioflavin S fluorescent microscopy.

Immunohistochemistry for TDP-43 revealed NCI, sparse dystrophic neurites, and NII in the neocortex. Phosphorylated tau immunohistochemistry did not reveal tufted astrocytes.

Case 5

The fixed left hemibrain weighed 510 grams. Macroscopic findings revealed no cortical atrophy in the neocortex. The medial temporal lobe was free of atrophy. Sequential sections revealed marked enlargement of the anterior and temporal horns. The hippocampal formation and amygdala had mild to moderate atrophy. The striatum and the subthalamic nucleus were unremarkable. The substantia nigra showed decreased pigmentation. The cerebellar sections showed no unusual features.

On hematoxylin and eosin-stained sections, there was mild neuronal loss and gliosis in the temporal cortex. The subthalamic nucleus had no neuronal loss. The substantia nigra showed mild neuronal loss.

Thioflavin-S fluorescent microscopy showed many SP, but no NFT in the neocortex. Sparse SP and a few NFT were detected in the hippocampus.

Immunohistochemistry for TDP-43 revealed many NCI in all cortical layers. The striatum and hippocampus had moderate NCI. Phosphorylated tau immunohistochemistry did not reveal tufted astrocytes.
