# Supplementary figures and images for: Frontotemporal Lobar degeneration with TDP-43 presenting as progressive supranuclear palsy syndrome
Source: Acta Neuropathol Commun. 2025 Jul 9;13:151. doi: 10.1186/s40478-025-02058-0 (PMC12239482; doi:10.1186/s40478-025-02058-0)

Supplemental Table. Clinical features of in 5 cases of FTLD-TDP-PSP


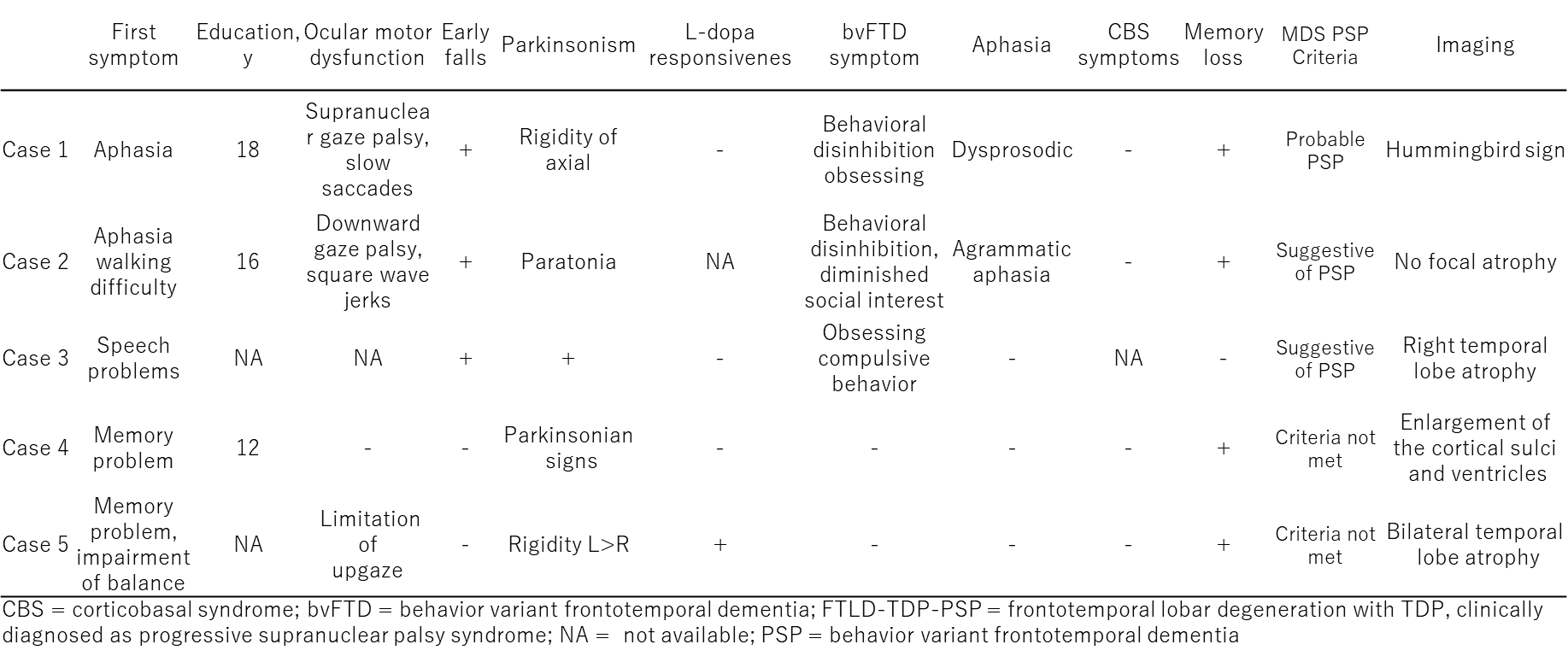

Supplement: Supplementary file 2 — Supplementary Material 2 [file 40478_2025_2058_MOESM2_ESM.docx]
